# Experiences and Perspectives of Traditional Bullying and Cyberbullying Among Adolescents in Mainland China-Implications for Policy

**DOI:** 10.3389/fpsyg.2021.672223

**Published:** 2021-07-06

**Authors:** Jiameng Li, Therese Hesketh

**Affiliations:** ^1^School of Medicine, School of Public Health, Center for Global Health, Zhejiang University, Hangzhou, China; ^2^Faculty of Population Health Sciences, Institute for Global Health, University College London, London, United Kingdom

**Keywords:** bullying, schools, psychosocial well-being index-short form, qualitative, China

## Abstract

The prevalence of traditional bullying and cyberbullying is high among Chinese adolescents. The aims of this study are to explore: (1) characteristics of children who are targets or perpetrators of traditional bullying or cyberbullying; (2) causes of bullying in middle school; (3) reactions and coping strategies of bullying victims; and (4) impacts of bullying on victims' psychosocial well-being. Students were selected based on the findings of previous quantitative research at schools in Zhejiang, Henan, and Chongqing. Snowball sampling led to identification of more informants. Semi-structured interviews were conducted with students involved in traditional bullying and cyberbullying as perpetrators, victims, and bystanders. Forty-one students aged 12–16 years (21 boys and 20 girls) from 16 schools in three provinces participated. Data collection and analysis followed a grounded theory approach. Among these students traditional bullying was much more common than cyberbullying, but there was a large overlap between the two types. The results informed a conceptual framework which identified the main causes of bullying in these settings: these included lack of education about bullying, inadequate classroom and dormitory management, and teachers' failure to recognize and punish bullying. Children with specific characteristics (such as being unattractive or low-achieving), were more likely to be bullied. Most victims lacked support of parents and teachers even when requested, leading to poor psychosocial well-being, difficulties with socialization, and poor academic performance. Our findings suggest that schools need to address bullying culture, through multi-faceted locally-appropriate approaches, based on zero tolerance. It is crucial to ensure that students, teachers and parents recognize the importance of such interventions.

## Introduction

In October 2019, a film about school bullying called Better Days was a box-office hit in China. Set in a Chinese high school, it tells the story of the aftermath of a girl's suicide. She had been relentlessly bullied and jumped to her death from a school balcony, as classmates photographed the event on their phones. The film highlighted the pervasive culture of bullying in many Chinese schools, and it resonated widely, sparking a national debate about bullying and its impacts in China for the first time. This film has led to calls to address the problem of bullying and its consequences as a matter of urgency (People's Daily, 2020).

The most widely accepted definition of bullying is that student is being bullied or victimized when he or she is exposed, repeatedly and over time, to negative actions on the part of one or more other students (Olweus, 1994). Negative actions can be carried out in the form of physical bullying, verbal bullying, spreading rumors, isolating, threatening, and damaging possessions (Kowalski and Limber, 2007; Hart et al., 2013). Cyberbullying is broadly defined as bullying performed *via* electronic means such as mobile/cell phones or the internet (Olweus, 2012). It includes: teasing/insulting online, spreading rumors online, exposing private information, and excluding from online groups (Smith et al., 2008; Huang and Chou, 2010). While there are clearly similarities between cyber and traditional bullying, there are also important differences, such as the identity of cyberbullying may be unknown; cyberbullying can happen anywhere and at any time; cyberbullying spreads faster and can reach larger audiences compared with traditional bullying (Slonje and Smith, 2008; Erdur-Baker, 2009).

Bullying has long been recognized as a serious problem among children and adolescents in many countries (Chan and Wong, 2015a). Research from the UK sampled 298,080 students aged 15 years reported that 27% had been involved in traditional bullying, and 3% reporting both traditional and cyberbullying (Przybylski and Bowes, 2017). A US study of 28,104 adolescents aged 14–17 years reported 23% had been victims of any form of bullying (cyber, relational, physical, and verbal) (Waasdorp and Bradshaw, 2015). A study among Australian adolescents aged 11–17 years in 2016 reported that the prevalence of traditional bullying victimization was 13.3% and perpetration 1.6% (Thomas et al., 2017). Bullying in Chinese societies can be viewed in the context of collectivism, which emphasizes maintaining interpersonal harmony and group conformity (Chan and Wong, 2015b). Bullying has often been seen as a collective conduct and social exclusion is a key form of school bullying in Chinese adolescents. Previous studies found both traditional bullying and cyberbullying to be common among Chinese children and adolescents. A study among 187,328 adolescents aged 13–17 years in 18 urban areas of mainland China showed that 66% of boys and 49% of girls had ever been bullied (Qiao et al., 2009). Another study of 3,774 middle school students in urban and rural areas of three provinces of China found 36% of participants were traditional bullying victims, 9.5% traditional bullying perpetrators, 31% cyberbullying victims, 17% cyberbullying perpetrators (Li et al., 2019).

Over the past few decades, it has been increasingly recognized that bullying is a serious threat to healthy child development (Smokowski and Kopasz, 2005). A growing body of evidence now confirms that being a target of bullying in childhood jeopardizes well-being and leads to mental health problems early in life (Ryu, 2014). Victims are a marginalized group at risk of negative social and emotional outcomes, such as social isolation, loneliness, low levels of perceived peer support, depression, anxiety, withdrawal, and insecurity (Nansel et al., 2001). A systematic review and meta-analysis, including papers from several countries, demonstrated the relationship between school bullying victimization and later health outcomes, such as depression, low self-esteem, and self-harm (Ttofi et al., 2011). A study of 1,225 American students aged 12–18 years reported that bullying victimization was associated with psychological symptoms, including getting worried, nervous, and scared easily (Hase et al., 2015). A bullying survey among 6,406 Chinese adolescents found that being a victim of any type of bullying was significantly associated with all kinds of mental health problems (Yen et al., 2014).

Although there is a large body of research examining the psychosocial impacts of bullying on victims, little has been conducted in mainland China, and very little has used qualitative methods. Qualitative research is especially valuable to enhance our understanding of children's experiences and perceptions of bullying, and to thereby inform policy. Therefore, this research utilizes in-depth interviews to investigate children's experiences and perceptions of bullying, as well as the effects on victims' psychosocial well-being, with a view to inform interventions. This qualitative study employs a grounded theory approach, in which data collection and analysis are conducted through the interactions among the interviewer, the research team and informants. Grounded theory approach is deemed most appropriate to allow participants' perspectives to emerge and to explore the complexity of a specific phenomenon (Mishna et al., 2009; Forsberg et al., 2014). According to grounded theory, data collection and analysis take place in parallel, and this iterative process guides the interviewers, which helps to make the interviews more focused over time (Thornberg et al., 2018).

Elsewhere and especially in western countries, many school-based programs have been implemented to reduce bullying. A recent review suggested that multi-faceted programs, combining different interventions were more effective. Such programs typically include punitive measures, involvement of parents, and strict playground supervision (Ttofi and Farrington, 2010). A meta-analysis of 13 studies found that the effectiveness of school-based anti-bullying programs was uncertain, and some programs were unsuccessful (Lee et al., 2015). An important predictor of success was the conditions surrounding implementation, including consistent support from school principals. Another study also reported the varying effects of whole-school anti-bullying programs and concluded that the effective ingredients were the intensity, duration, and implementation fidelity of the programs (Menesini and Salmivalli, 2017). In recent years, a number of severe bullying cases among Chinese adolescents have been reported by the media, attracting considerable attention. In 2016, the Chinese Ministry of Education introduced some policy initiatives aimed at the prevention and management of school bullying (Ministry of Education, 2017). But the continued high prevalence of bullying in schools in China illustrates that we need to understand more about bullying to inform effective measures.

This research therefore aimed to explore the following areas:

1. the characteristics of children who are either targets or perpetrators of traditional or cyberbullying
2. the causes of bullying within a middle school context
3. reactions and coping strategies of victims of bullying
4. long-term impacts of bullying on victims' psychosocial well-being.

## Methods

### Sample

This qualitative study employed a grounded theory approach to the collection and analysis of semi-structured interviews with adolescent students aged 12–16 years, who had experience of traditional school bullying or cyberbullying as perpetrators, victims or bystanders. Screening questions were asked to identify whether informants with experience of traditional school bullying (1. physical bullying-hitting, kicking, beating; 2. verbal bullying-mocking, ridiculing; 3. spreading rumors; 4. exclusion/isolation; 5. threats; 6. damage to possessions) or cyberbullying (1. teasing/insulting online; 2. online spread of rumors 3. exposure of private information online; 4. exclusion from online groups; 5. online threats). These same 11 items generated more questions. For example, with regard to physical bullying the questions were: “Have you bullied others physically in school in last year?” “Have you been bullied physically in school in last year?” “Have you ever seen others being bullied physically in school in last year?”. This study was carried-out in the same schools where we had previously conducted a quantitative survey about bullying (Li et al., 2019), so we had gained familiarity with the school settings. Teachers at the schools helped to identify students, who had been involved in bullying, for inclusion in the qualitative study. Snowball sampling led to identification of more informants. Forty-one students (21 boys and 20 girls) aged 12–16 years were found to be suitable to participate in the study. They were from 16 schools in urban and rural areas of Zhejiang, Chongqing, and Henan provinces. Zhejiang is a high income eastern coastal province with a population of 64 million, Henan is a lower middle-income province with a population of 99 million, and Chongqing is a higher middle-income municipality with a population of 32 million. The informants came from across the range of socio-economic backgrounds. The 16 schools included 9 public and 2 private schools in urban areas, and 2 county schools and 3 township schools in rural areas. Eleven of the schools were predominantly boarding schools, with students mostly staying at school from Monday to Friday, and returning home at weekends, because the schools are located too far from students' homes for daily attendance.

### Procedure

Interviews were conducted from July to August, and in December 2019. Interviews were conducted in Mandarin in Zhejiang and Chongqing, and in the local dialect in Henan. Interviews were held in a private setting in the schools. The average duration of the interview was about 50 min (with a range from 30 to 110 min). All of the interviews started with chatting about topics such as hobbies or gossip about favorite film stars, so informants would be relaxed before starting the interview. Data saturation refers to the point at which properties of a category or theme are no longer being modified by incoming data (Gleaser and Strauss, 1967). Empirical efforts have led researchers to suggest that data saturation occurs when incoming information results in minimal or no changes to the codebook. The codebook was developed recursively until no new preliminary or core categories were identified from new interviews (Wood et al., 2017). This was achieved after 35 of the 41 interviews indicating that our original selected number of participants was sufficient to reach data saturation.

Interviews were audio-recorded and transcribed verbatim with verbal informed consent from the participants. Observational notes were also made regarding informants' facial expressions. Findings from our quantitative study identified risk factors involved in bullying, such as boarding, poor academic performance and having a poor relationship with parents. We also found that bullying victims had significantly higher risk of psychosocial problems. Therefore, the interview outline was designed based on the findings from our previous quantitative study (Li et al., 2019). The main topics included: school life, such as classroom and dormitory management; experience of bullying as perpetrators, victims, and witnesses; reasons that bullying happens; characteristics of perpetrators and victims; reactions to bullying; whether victims seek help and how; psychosocial effects of bullying; and comments on existing school policies of bullying.

### Ethics

Ethical approval was obtained from the Ethics Committee of Zhejiang University School of Public Health before conducting the survey. The study background, content, and the interview purpose were explained to all participants and informed written consent was obtained. Informants were assured that the interview could pause or stop at any time, if they felt uncomfortable to continue. At the end of each interview, the contact information of the researchers was provided to informants in case they needed help or advice. None of the informants took-up the offer. Interview audio recordings were stored anonymously and confidentially in a coded disk.

### Data Analysis

Data were gathered and analyzed in an iterative process, resulting in revisions, including modifications to interview questions throughout the research process. The data analysis followed a grounded theory approach (Corbin and Anselm, 2008a). First, all transcript data were open coded to define the preliminary categories. Notes were taken to generate explanations of the emerging concepts, and to further develop the key categories, as well as to define the relationships between them. Second, in the focused coding phase, the most significant and frequent codes from the initial coding were used, resulting in a more focused and conceptual analytic approach (Forsberg et al., 2014). Lastly, we employed theoretical coding, referring to the process of selection of one or more core categories intended to generate a theory that connects the categories (Corbin and Anselm, 2008b). A theory about being bullied and associated poor psychosocial outcome, eventually emerged.

## Results

The sociodemographic information of the informants is listed in Table 1. Of the 41 informants in the sample, nine admitted they had ever bullied others in school, 17 reported having been bullied in school, nine stated they were both bullies and victims of traditional bullying, and six were bystanders who had witnessed bullying incidents. A further seven informants reported they were cyberbully victims and five described the cyberbully experience they had witnessed.

**Table 1:** Sociodemographic information of informants.

**No**.	**Gender**	**Age**	**Location of school**	**Boarding status**	**Grade**	**Bullying involvement**
1	Male	15	Urban	No	9th	bystander
2	Female	14	Rural	No	8th	bystander
3	Male	13	Rural	No	7th	Bully and victim in school
4	Female	14	Rural	No	8th	Victim in school
5	Male	15	Rural	No	9th	Bully in school
6	Male	14	Urban	Yes	9th	Bully in school
7	Female	16	Urban	Yes	9th	Victim in school
8	Female	15	Urban	Yes	9th	Victim in school
9	Male	14	Urban	Yes	8th	Bystander in school and online victim
10	Male	15	urban	Yes	9th	Victim in school
11	Male	13	Urban	Yes	7th	Bully in school
12	Male	13	Rural	No	7th	Bully in school
13	Male	14	Rural	No	7th	Bully and victim in school
14	Female	14	Urban	Yes	9th	Bully in school
15	Female	14	Rural	Yes	8th	Victim in school
16	Female	14	Rural	Yes	8th	Victim in school
17	Male	15	Urban	Yes	8th	Bully in school and online victim
18	Female	16	Urban	Yes	10th	Bully and victim in school
19	Male	15	Urban	No	9th	Bully and victim in school
20	Female	15	Urban	No	9th	Bystander
21	Female	13	Urban	No	7th	Bully in school
22	Female	13	Urban	Yes	7th	Bully in school
23	Female	13	Rural	Yes	7th	Bully and victim in school
24	Female	13	Urban	No	7th	Bystander
25	Male	15	Urban	No	9th	Victim in school; online bully and victim
26	Male	14	Rural	Yes	8th	Victim in school
27	Female	13	Rural	Yes	7th	Bully and victim in school
28	Female	13	Rural	Yes	7th	Bully and victim in school
29	Male	16	Rural	Yes	10th	Victim in school
30	Male	13	Urban	Yes	7th	Victim in school
31	Male	13	Rural	Yes	8th	Victim both in school and online
32	Female	12	Rural	Yes	7th	Victim both in school and online
33	Female	15	Rural	No	9th	Victim both in school and online
34	Male	12	Urban	No	7th	Bully and victim in school
35	Male	12	Urban	No	7th	Victim both in school and online
36	Female	15	Rural	Yes	9th	Bystander
37	Female	15	Rural	Yes	9th	Bully in school
38	Female	14	Rural	No	9th	Victim in school
39	Male	15	Rural	No	9th	Victim in school
40	Male	12	Rural	Yes	7th	Victim in school
41	Male	15	Rural	No	9th	Bully and victim in school

**Figure 1:**
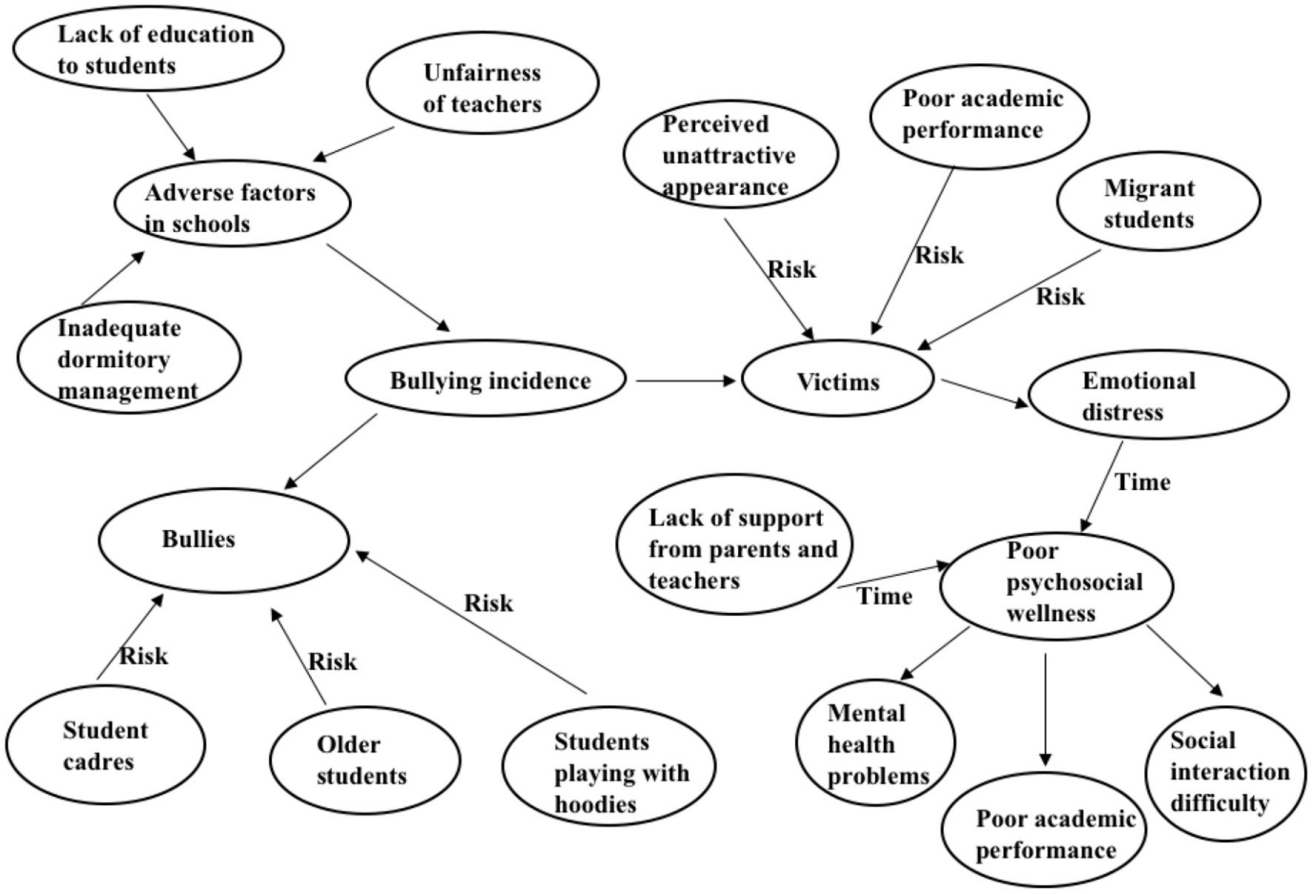
Conceptual framework of the poor psychosocial well-being of bullying victims.

Eight main themes were identified from the interviews: (1) Varied experiences of bullying. (2) Adverse factors in the school context. (3) Characteristics of bullies. (4) Characteristics of victims. (5) Emotional distress of being bullied. (6) Lack of support from parents and teachers. (7) Psychosocial problems related to bullying. (8) School responses to bullying. The grounded theory paradigm and the results of this study informed a conceptual framework, which is shown in Figure 1.

### Theme 1: Experiences of Bullying Were Very Varied

In terms of traditional school bullying, almost all the informants mentioned verbal bullying, such as teasing, calling nicknames or insulting. A 15-year-old boy, #5, said: “I did something really mean to my classmates. I read their scores out and teased them when they did not do well in exams. I saw they were really embarrassed.” A 14-year-old girl, #16, reported: “From 7th grade, some of my friends called me ‘little fattie'. At first, I felt annoyed, but now I don't care.” Social isolation was more common among girls. A 13-year-old girl, #21, said: “Last term, several girls asked me not to play with another girl. I didn't know why, but I did what they said and didn't talk to the girl when she came up to me. I saw she was upset but I didn't really care that much.” Rumor-spreading was usually related to relationships between girls and boys. A 14-year-old boy, #26, reported: “Someone spread rumors about me that I had fallen in love with a girl, which was really embarrassing.” Physical bullying seemed to be less common than verbal bullying. A 14-year-old girl, #4, said: “Schools don't allow fighting, so there isn't much physical bullying.” When fighting occurred, our informants said this was always among boys. A 13-year-old boy, #11, said: “Once during the afternoon nap time, a boy in my dorm kept talking. He wouldn't stop when I asked him. So I got angry and beat him.” Threats and forcing others to do certain things were common. A 14-year-old boy, #13, said: “After the afternoon nap, there is always a long queue for fetching water. Two boys always force others to get water for them, because they don't want to stand in the queue.”

Online verbal bullying was reported to be quite common by some informants. A 15-year-old boy, #1, said: “It is very common for us to use rude or dirty language online.” Exposing others' private information was another kind of cyberbullying. A 13-year-old girl, #24, revealed: “A girl refused to be the girlfriend of a boy, so the boy got angry. He insulted the girl and posted her private information online and asked his friends to share it around with others.” Exclusion was described as occurring in multi-player online games, because of selection of the best players for the team in order to win. Thus, the lower level players would be excluded. A 15-year-old boy, #17, said: “I am at a lower level, so my friends won't let me on to their team. I am very upset about this.”

There were mixed views about the relative prevalence of traditional and cyberbullying. Although cyberbullying often accompanied traditional bullying, the majority of informants stated that cyberbullying was much less common than traditional bullying, not least because phones were not allowed in almost all of the schools. A 15-year-old boy, #25, said: “Cyberbullying is not a big deal compared to traditional bullying. Cyber bullies may be the victims of traditional bullying in real life. In the cyber world, they have the chance to bully back, which releases the pressure of being bullied at school.”

But some informants thought cyberbullying was common. For example, a 13-year-old girl, #24, reported: “I think cyberbullying is much more common around me—our school has good measures to prevent school bullying, and there is little physical bullying in my school. Schools aren't really aware of cyberbullying, so they don't have rules about it. Although schools don't allow us to carry smartphones, we just hide them and carry them around with us.” A 13-year-old boy, #30, stated: “In school we study all the time even during the class break, so there is no chance to bully. Lots of my classmates are quiet and shy, but they are very active in the cyber world. So there isn't much bullying in school, but in the Chat group of our class, there's lots of cyberbullying.”

### Theme 2: Adverse Factors Within School Context

#### Lack of Awareness and Effective Education About Bullying

The informants pointed out a number of factors which contribute to bullying in the school environment. First, there is very little education about bullying. A 16-year-old girl, #7, said: “There is little education for students about bullying, especially about cyberbullying. We always learn about school bullying incidents from gossip, but never from teachers. Schools don't want negative news to spread, so our teachers are required to hide anything bad and don't tell us what happens. I think teachers should share the news with us and teach us what to do when bullying happens.” A 15-year-old boy, #17, said: “Several months ago, a boy died in school. Some people said he was beaten to death by a group of boys, while some others said he committed suicide. We didn't know the truth until now and the headteacher just asked us not to discuss this whether in school or online and not to spread rumors because this would harm the reputation of our school.” Very occasionally, classes on bullying are provided, but they are very didactic, and not taken seriously. A 15-year-old boy, #41, said: “We had a lecture about school bullying and cyberbullying, but I just didn't listen and most of my classmates didn't pay attention. Teachers didn't care whether we were listening because they didn't think it's important and just wanted to finish the task, because the school requires it.”

#### Inadequate Dormitory Management

In the boarding schools, dormitory management is a challenge. Some boarding schools don't employ dormitory “keepers,” so no one takes care of students when they are in the dormitory. In other schools, retired soldiers are hired as “dormitory keepers” to discipline children, make sure the dormitory environment is safe and that bullying is controlled. But several informants complained about their unqualified dormitory keepers and poor dormitory discipline. A 15-year-old boy, #10, said: “Dorm attendants are very mean and vulgar, and they even use corporal punishment. This is really horrible for the students.” Another 14-year-old boy, #6 stated: “The dorm keeper is really not responsible, and doesn't keep good dorm discipline. Sixteen students share one room and some of them talk until late at night, so I can't sleep. Sometimes there is bullying in dorms, but dorm keepers don't do anything about it.”

#### Unfairness of Teachers

Teachers may have a preference for some students and thus are prone to favor them. For example, a 13-year-old boy, #31, reported: “The headteacher really likes a boy in my class They have a very good relationship. So when this boy bullies others the headteacher just ignores it.” Informants also reported that teachers display preferences for top students and would take the side of the top students in situations of conflict. A 15-year-old boy, #17, said: “Two of the top students often bully a particular boy. Once they played a horrible joke on him, so he was really distressed and reported it to the headteacher. But the headteacher did nothing, because he naturally took the side of the top students.”

### Theme 3: Characteristics of Bullies

#### Student Cadres

During the interview, a number of informants said that the worst bullies were Chinese student cadres. Cadres are among the best students, have some responsibilities in the class and represent teachers, in their absence. Therefore, they have power, which they are known to misuse. A15-year-old girl, #8, stated: “Cadres are always good students, who sit in the front of class. They always look down on bad students, saying mean things to them.” A13-year-old girl, #23, said: “I have conflicts with a student cadre, because I tell him he acts unfairly. So he always picks on me and reports on me for stupid things, like that I am not listening in class.”

#### Older Students

The age range is 4 years among middle school students. All students in the same school share play areas, sport facilities, canteens and school shops. Older students are reported to bully younger ones. A 13-year-old girl, #27, reported: “It often happens that when I haven't finished my lunch, I am forced to give up my seat by the higher grade girls.” A 14-year-old boy, #13, said: “When I was in 7th grade, the 8th and 9th grade students used to take our bats when we were playing ping-pong. There is no way that 7th grade students can get to play ping-pong.”

#### Students Keeping Relationships With Chinese “Hoodies”

Chinese hoodies are adolescents who have dropped out of school, and hang around on the street, and are often involved in antisocial behavior and even petty crime. But they are viewed by many school students as “cool” and some students cultivate relationships with these hoodies. These relationships increase the status of these students, who then feel they can bully other students. This phenomenon was reported by a number of informants across the three provinces. A 12-year-old girl, #32, said: “My girlfriend, S, has good relationships with these boys. Another girl, C, spread rumors that S had slept with these boys. Once on the street, S and her hoody friends slapped C on the face 50 times and filmed it, spreading it to lots of people through social media.”

### Theme 4: Characteristics of Victims

#### Perceived Unattractive Appearance

During the interview, the informants said that being “short and skinny” was a particular risk for bullying of males; whereas, for female students, it was being “fat and unattractive.” A 14-year-old boy, #6, said: “I always tease short guys in my class, and others do too. I don't think this is bullying.” A 15-year-old boy, #25, who is very thin and short, reported: “There is a big guy in class, he always bullies me by dragging my collar and throwing me around for fun.” A 14-year-old girl, #2, stated: “There is a girl in my class, who is always teased and has almost no friends because she is so fat and ugly. Also she doesn't change her clothes much.”

#### Poor Academic Performance

Some informants in the interviews also talked about such things. A 12-year-old boy, #40, said: “Students at the bottom of the class are often teased for having something wrong with their brain.” Similarly, a 13-year-old boy, #11, reported: “There is a boy at the bottom of my class, who looks stupid. He is always insulted and beaten by others, but he never fights back, and never reports this to teachers.” Not retaliating or not fighting back also made students more likely to be bullied. A 13-year-old girl, #23, stated: “A girl in my class is often bullied, but never retaliates. Besides, she is ugly and at the bottom of the class, so no one likes her anyway.”

#### Migrant Students

In China internal migration for work is common, and some children accompany their migrant parents from rural to urban areas. This often causes difficulties with assimilation into local schools because students exclude the newcomer. For example, a 13-year-old boy, #31, reported: “My parents are migrant workers, and I migrated with them when I was in 2nd grade. Since then, I have been bullied by my classmates, who constantly harass and bully me and the other migrant students. Once they even put tadpoles in my cup. I want to make friends with the local students, so I share my secrets with them, but they just spread this everywhere, and joke about this. Almost no one plays with me. I feel I am not as good as the others. I get really sad.” Teachers were reported as being aware of this treatment of migrant students, but that they did nothing to protect them. A recent migrant, #31, said: “Teachers never care about us migrant students, and they don't pick on us to answer the questions even when we raise our hands. They don't praise us when we perform well, but they do blame us as soon as something goes wrong.” A 15-year-old boy, #39, stated: “I migrated to the city, and started at this school in the city. I was new here and not familiar with anything. One day, a boy in my class lost his toy, a spinning top. He accused me of stealing it, and didn't believe me when I denied it. He and his friends poured water on my textbooks and bullied me all the time. I really couldn't focus on studying after that horrible experience.”

### Theme 5: Negative Feelings and Emotional Distress

Children spoke openly about their emotional distress at being bullied. After being hit, punched and insulted by a boy, a 16-year-old girl, #7, said: “I was in pain, very angry and hated that boy so much.” Anger, sadness, and embarrassment were all reported by victims. It was notable that four informants cried during the interview when asked about their feelings. For example, a 15-year-old boy, #10, reported an event which occurred 2 years previously, “I was teased by a boy. The name of the main character in a book we read in class was the same as my mother's. This boy made rude jokes, humiliating my mother and me. I was very hurt and upset.” Cyberbullying was also hurtful. A12-year-old girl, #32, said: “Two girls from higher grades insulted me on WeChat and constantly sent warning messages that they would beat me. I was really afraid to be alone, in case they came and got me.”

However, a minority of informants appeared to have become used to the bullying culture and were able to virtually ignore it. A 15-year-old boy, #25, reported: “I don't care about being bullied because I'm always being treated like that. I tell myself I have to bear this- so I do.”

### Theme 6: Lack of Support From Teachers and Parents

Some informants talked about the way that teachers and parents were unaware or deliberately ignored the situation. Some informants did not want to bother parents or teachers. A 14-year-old boy, #13, said: “I don't report bullying, because it is really common. There is no need to report it.” A 16-year-old girl, #7, said: “I don't report to teachers, because they are busy. I don't tell my mother because she will worry about me.” Some thought the intervention of adults would make it even more difficult to get along with peers. A 12-year-old boy, #35, reported: “I haven't told my parents, because they would report to the head teacher, and I don't want my teachers to know, because it would make my classmates hate me.”

Some informants could not tolerate long-term bullying and did ask for help from adults. The reaction of parents varied considerably. Some parents were sympathetic, but others thought bullying was nothing serious or even quite normal, which sometimes made things worse. A 15-year-old girl, #8, said: “A is the head girl and I don't know why I upset her. She asked all the others not to play with me. I told my mother, but my mother didn't think it mattered. She said ‘you can play by yourself if there is no one to play with you.' I also asked my brother to help me. However, no one could understand why I was so upset, and they all thought there was no issue. The fact is it has made me cry almost every day.”

Although a few teachers really helped in bullying situations, most did not provide support. A 13-year-old boy from a migrant family, #31, reported: “My parents came to school to complain, but the teachers didn't help. The local students still bullied me, so my parents sent me to learn martial arts, so that I could protect myself. Three years later, I was strong enough, and now no one dares to bully me.”

### Theme 7: Poor Psychosocial Well-Being of the Victims

#### Mental Health Problems

Some informants described experiencing multiple forms of bullying, including rejection by classmates, verbal bullying and physical bullying for an extended period of time. As a result, negative emotions appear to accumulate, leading to mental health problems. For example, a 15-year-old girl, #33, experiencing rejection and verbal bullying both in school and online, reported: “I have been bullied all the time since primary school. I think I am too stupid, so they all dislike me, and no one hangs out with me. I do everything on my own, so I feel really lonely. I became depressed and quit school for a while. I have been taking anti-depressants for more than a year. Even now, some classmates call me ‘ghost' and shout out ‘the ghost is coming' when I pass by. They always insult me *via* WeChat and QQ.” A 15-year-old girl, #7, had experienced different kinds of bullying for many years and had harmed herself with cutting of her forearms. She said: “At first, I hated myself because I was near the bottom of class, and they bullied me. Gradually, I got used to being bullied and now I've become numb, I just feel inferior to everyone else.”

#### Poor Academic Performance

Some of the informants who experienced bullying said it affected their interest in studying and their academic results. A 13-year-old boy, #31, reported: “Being bullied has made me really sad. Before I was bullied, I was a top student, but now I can't concentrate on my study, because they bully me in different ways both in school and online all the time. Eventually, I lost interest in studying altogether, and my marks have got worse and worse.”

#### Social Interaction Weakening

Long-term bullying and social rejection discouraged children from actively making friends, which also compromised psychosocial well-being. A 15-year-old girl, #8, described this well: “I was isolated and betrayed even by my best friends in the last term. It was really hurtful and I cried a lot. From then on, I didn't dare to try to make friends. Only if others reach-out to me, will I be able to make friends again. I am afraid of being hated.”

### Theme 8: School Measures for Bullying

Many informants reported that schools installed cameras in classrooms and corridors, which may have helped to prevent some forms of bullying. A 14-year-old girl, #2, said: “Dormitories, playgrounds and school shops don't have cameras, so that's where bullying may happen.” Some informants said school safety was sometimes mentioned in the weekly class meeting. A 13-year-old girl, #21, reported: “School bullying is mentioned when the class meeting is about school safety, but with very few details.”

According to most informants, there were policies and punishments for bullying, such as criticism, warning, suspension or expulsion of bullies, according to the severity of the incident. But in some schools, they had some distinct measures to prevent children from being involved in bullying. For example, a 13-year-old boy, #31, reported: “Reports of bullying are recorded in the personal records, which accompany us all our life. So this is a good way to deter students from bullying.” A 15-year-old girl, #36, stated: “Students who have records of misbehavior and bullying sometimes aren't allowed to sit public examinations like high school entrance examinations until the record is removed.” In her school, there is an “anonymous box” for reporting misbehaviors. She said: “We have an anonymous mailbox in our school, which provides space for anonymous reporting of other students' misbehavior. These messages are read by school leaders and it is a good way to report something bad that you have seen, and which you don't dare to speak out about.”

## Discussion

To our knowledge, this is the first study involving in-depth interviews about both traditional bullying and cyberbullying among middle school students in mainland China. Our findings shed light on several areas: (1) adverse school factors such as unfairness of teachers, and inadequate dormitory and classroom management contribute to the school bullying culture; (2) victims of bullying do not get enough support and help from adults; (3) lack of support and prolonged bullying lead to mental health problems, difficulties with socialization, and poor academic performance, in victims, as shown in the framework in Figure 1.

Our study shows that bullying was a normal occurrence and there was an overall tolerance of bullying in these schools. Even for bystanders, most of them didn't intervene to help the victims. A study reported that there were two main bystander feelings: (1) bystander fear of getting attacked or bullied themselves or losing social status if they defended the victim deterred defending behavior, and (2) moral feelings that motivated defending (Forsberg et al., 2018). In hypothetical scenarios, participants usually approve of the behavior of witnesses who defend the victims, and disapprove of those who assist bullies or ignore obvious bullying (Gini et al., 2008). In our study, bullying of unattractive or less academic students seemed to be widely accepted. Perceived physical attractiveness has been shown to elicit preferential treatment from others and less attractive children are less accepted by peers (Vannatta et al., 2009). The bullies themselves were reticent about sharing their experiences, providing very little detail, even with specific probing and a non-judgmental approach from the interviewer, suggesting that bullies may be aware of the harm to their victims, and may feel ashamed of their actions.

There was considerable overlap between traditional bullying and cyberbullying: of the seven cyberbullying victims, five were also victims of traditional bullying; of the 26 traditional bullying victims, five were cyberbullying victims. The small number of cyberbullying reports compared to traditional bullying in our study and the overlap between them suggest that in these schools cyberbullying creates relatively few new victims and cyberbullying victims are to a large degree the same as those victims of traditional bullying. One explanation for the small number of cyberbullying victims is the limitation in use of mobile phones in school. The overlap may be because the characteristics that make adolescents vulnerable to traditional bullying may be the same as those for cyberbullying (Hinduja and Patchin, 2008). This overlap has been observed in other studies. A study including 120,000 adolescents aged 15 year-old in England found that most cybervictimization occurred alongside traditional bullying and very few victims only experienced cyberbullying (Wolke, 2017). Another study among 4,000 adolescents in South Korea also showed a strong overlap between cyberbullying and traditional bullying, and reported that cyberbullying should be regarded as part of a general pattern of traditional bullying (Lee and Shin, 2017). However, other study found that cyberbullying victimization is different from the victimization of traditional bullying, and they are weakly related to each other (Dempsey et al., 2009).

In our conceptual framework, aspects of the school context, such as classroom or dormitory management, student-teacher interactions, and teachers' reactions to bullying are all important factors affecting school bullying culture. According to informants, teachers are often unwilling to intervene. Thus, teachers may actually be contributing to the bullying culture, because of failing to speak out against bullying behaviors, a phenomenon described elsewhere (Espelage and Swearer, 2003). This may be especially the case for top students who bully others. Within the Chinese cultural context of emphasis on academic achievement, most teachers treat high performing children preferentially. Teachers tend to attribute good characteristics to children who do well academically, and assume they won't bully others (Fox and Boulton, 2005). This is often referred to as “the halo effect:” positive qualities are more likely to be attributed to attractive individuals, whereas behaviors incongruent with those qualities are overlooked or judged more mildly. Therefore, bullying is more likely to go undetected and unpunished when carried-out by students who are favored by teachers (Marucci et al., 2021). The halo effect implies that the better a student's performance in academia, the better the teacher's judgement in his/her other performances (Dompnier et al., 2006). A study among Chinese primary school students showed that sometimes teachers may be the source of school bullying when they show prejudice against an individual student, and 28% of the participants thought that students disliked by teachers were more likely to be bullied because they were more likely to be seen as an easy target (Ma and Chen, 2017). In our study, a number of participants reported that student cadres were perpetrators, and 943 (26%) of the participants in our quantitative study reported they had ever been bullied by student cadres. Student cadres are empowered by teachers to manage other students, and at the same time they are required to help other students. However, many teachers favor the cadres, contributing to the social hierarchy within the class (Liu, 2017). The resulting power imbalance between the cadres and other students is an important contributor to a bullying culture.

As indicated in our framework, bullying victims may lack support from others. In our schools bullying was virtually normalized, so few students bothered to report it, a phenomenon reported elsewhere (DeLara, 2012). Most of the victims did not tell parents or teachers that they had been bullied for fear of being ignored or not taken seriously. A study among adolescents aged 14–18 in the U.S found that children did not disclose bullying to adults if they believed there would be no appropriate response (DeLara, 2012). So the bullying victims in many settings do not get the help and support they need from adults. Lack of adult intervention means children are more likely to be bullied repeatedly with adverse effects on children's psychosocial well-being (Chan and Wong, 2015b). A study among children aged 9–14 years in the UK found that support from teachers, friends and family members was the most effective strategy to overcome negative emotions associated with bullying (Hunter et al., 2004).

Bullying victims may have psychosocial problems, such as mental health symptoms, difficulties with socialization, and poor academic performance, as shown in Figure 1. The link between bullying victimization and poor mental health, especially depression and anxiety, is well-established (Evans et al., 2014; Thomas et al., 2017). As the great majority of cyberbullying victims are also bullied in traditional ways, it is difficult to know to what extent psychological problems can be attributed to cyberbullying (Olweus, 2012). Even so, cyberbullying seems to be less harmful compared to traditional bullying according to our informants. Cyberbullying is often motivated by negative emotions such as anger or revenge in real life, and it may provide an outlet to alleviate negative emotions generated by traditional bullying (Paez, 2018). The previously mentioned study of 120,000 adolescents aged 15 in England reported that a much higher percentage of variance in poor mental well-being (5% of well-being variability) was explained by traditional bullying compared with cybervictimization (<1%) (Wolke, 2017). This aligns with our findings that traditional bullying may be a more important risk factor for mental health problems than cyberbullying.

Consistent with previous studies, we found most of the bullying victims have experienced emotional distress, including anger, anxiety, sadness, and extreme embarrassment. Most of the victims seem to have developed a sense of inferiority, and low self-esteem. Our informants indicated that repeated bullying over a long time was a particular cause of mental health problems due to the accumulation of emotional difficulties. This has been shown elsewhere: a longitudinal study among adolescents aged 12–14 years in the US showed that prolonged exposure to bullying increases the likelihood of poor mental health outcomes (Haddow, 2006). Another study among Norwegian adolescents aged 13–15 years found a dose-response relationship between bullying victimization and negative mental health outcomes (Natvig et al., 2001).

Our study found social interaction difficulties and poor social skills in bullying victims, especially in those excluded by peers. Social exclusion is observed as a particular school bullying issue in Chinese societies (Chan and Wong, 2015a), and several of our informants talked about this and described difficulties in making friends. A study among children aged 9–11 years in the UK suggested that victims of school bullying were perceived to be socially unskilled by teachers, peers and themselves (Fox and Boulton, 2005). Bullied children showed less understanding social interactions, leading to inappropriate or odd social behavior (D'Andrea et al., 2012), which in turn, leads to a higher risk of being bullied or isolated. Improvement of social skills of victims thus could be a useful intervention to reduce vulnerability to being bullied. Generally individuals with good social skills make and maintain friends more easily, and can better deal with interpersonal problems (Silva et al., 2018). Social skill training is important in anti-bullying programs, which includes teaching good manners, making friends, empathy, self-control, emotional expressiveness, assertiveness, and solution of interpersonal problems (Lee et al., 2015).

In traditional Chinese culture, great importance is attached to academic achievement which is closely linked to future financial success and higher social status (Hesketh and Qu, 2005). So the fact that children in our study who experienced prolonged bullying could not focus on their studies is a particular concern. A longitudinal study among middle school students in the US found that students who were bullied were likely to fall into lower academic rank, receive lower grades and engage less in academic tasks (Juvonen et al., 2011). A systematic review identified that bullying victims experience distress such as depression, loneliness, and anxiety, and this has a negative influence on academic performance (Nakamoto and Schwartz, 2010). Therefore, when assessing children whose academic performance has declined, the possibility that they are being bullied should be considered. We also found that students with poor academic performance were more likely to be bullied, causing a vicious cycle. Peer victimization acts as both a cause and an outcome of poor academic performance.

Our study highlights implications for policy. School policies in most schools in China are about punishment, with no integration of bullying intervention programs in school activities, and no involvement of parents. But there are now lessons from successful interventions elsewhere. For example, the Olweus Bullying Prevention Program, initially implemented in Norway, is the most widely recognized program for addressing bullying, and it incorporates a comprehensive school-wide approach, including training all school staff in bullying prevention, enforcing clear rules and consequences related to bullying, involving children in regular discussions about bullying, and strengthening parents-school connection to support the program implementation (Smokowski and Kopasz, 2005). The successful KiVa program from Finland has been duplicated in several countries. This comprehensive multi-faceted program includes classroom discussions, short films of bullying, and role-playing exercises. Teachers are trained and issued with special vests to wear to enhance their visibility in the playground and parents are given guidance and advice about bullying indications (Ttofi and Farrington, 2010; Van der Ploeg et al., 2016). In China, there has been some recent progress. Drawing on experience from other countries, researchers at Shandong Normal University have reduced bullying by 50% in participating schools. Drawing on measures from the KiVa program, they also developed some locally appropriate measures, such as creating student peer groups to stop bullying and support victims, and working with the police to deter hoodies from hanging around outside schools and encouraging bullying (Zhang, 2017). Lessons from our findings and the evidence from elsewhere, suggest that multi-faceted locally-appropriate approaches, should be taken to address the issue of endemic bullying in schools. These include: (1) The need for schools to acknowledge the existence of the bullying culture, that can be replaced with the zero-tolerance approach. Students, teachers and parents must all be made aware of the zero-tolerance culture, and students must be told that bullying will result in punishment. Posters explaining this should be posted around the school. At the beginning of school terms teaching about zero-tolerance, and the adverse effects of bullying should be held for all classes. (2) Schools should strengthen supervision in high risk spots such as playgrounds and school shops, where many of our informants mentioned bullying was especially common. This “hot-spot” supervision has been found to be an effective intervention in a number of settings (Gaffney et al., 2019). (3) Dormitory keepers should be selected carefully and be provided with specific guidance about acceptable dormitory behavior, with a system of rewards and punishments to motivate better behavior. (4) Teachers need to act on bullying incidents and foster positive relations with students by treating all children equally (McKenzie, 2009; Bibou-Nakou et al., 2012). (5) Parents need to be made aware of bullying and its dangers, and need information about how to support their children if they experience bullying. (6) Schools need to work with police, where necessary, to deter hoodies from hanging around schools and causing bullying incidents.

## Limitations

There are several limitations in this study. (1) Selection bias is a clear limitation. Students with very unpleasant experience of bullying, or who were bullies themselves, may have refused to participate. (2) There may be a tendency to social desirability bias, especially given the sensitivity of the issues. Despite our efforts to build trust during the conversations, some participants may not have fully expressed their feelings in the context of a research interview and a few participants initially spoke in the third person, but not in the first person to disclose their own stories, perhaps suggesting the experiences were difficult to talk about or admit to. The latter is probably especially true of perpetrators. (3) Another possible limitation was the use of interviewing as the sole method of data collection. Information elicited through a single interview could be limited by factors such as a student's current emotional state, level of comfort with the interviewer, desire to provide expected responses, and ability to quickly think about and verbalize complex issues. Therefore, what students report may not reflect the actual situations. Future research should use diverse research methods (e.g., multi-session interviews, focus groups, observations) from several sources, including parents and teachers.

## Conclusion

Our findings suggest: (1) adverse school factors such as unfairness of teachers, and inadequate dormitory and classroom management contribute to the school bullying culture; (2) victims do not get enough support and help from adults; (3) lack of support and the prolonged bullying lead to psychosocial problems among bullying victims, including mental health symptoms, difficulties with socialization, and poor academic performance. The fact that bullying is now being more openly discussed in society is helping to raise awareness about the need to address it. Schools need to take responsibility through zero tolerance approaches involving students, teachers, and parents, and ensuring that the importance of such interventions is understood.

## Data Availability Statement

The raw data supporting the conclusions of this article will be made available by the authors, without undue reservation.

## Ethics Statement

The studies involving human participants were reviewed and approved by Ethics Committee of Zhejiang University School of Public Health. Written informed consent to participate in this study was provided by the participants' legal guardian/next of kin.

## Author Contributions

TH and JL designed the study. JL conducted data collection and analysis and wrote the draft. TH revised it, back, and forth several times. Both authors contributed to the article and approved the submitted version.

## Conflict of Interest

The authors declare that the research was conducted in the absence of any commercial or financial relationships that could be construed as a potential conflict of interest.
