# Supplementary material for: Experiences and Perspectives of Traditional Bullying and Cyberbullying Among Adolescents in Mainland China-Implications for Policy
Source: Front Psychol. 2021 Jul 6;12:672223. doi: 10.3389/fpsyg.2021.672223 (PMC8290073; doi:10.3389/fpsyg.2021.672223)
Supplement: Supplementary file 1 [file Table_1.DOCX]

**Questions for School Bullying**

**-Basic information**

1. Tell me about your family (number of siblings, whether live with parents, relationship with parents).

2. How long have you attended this school? How about school? (students’ perception of school climate, order and discipline; school/class size; relationship with teachers/classmates)

3. How about your academic performance? Do you know the rank?

4. Sometimes kids pick on each other; sometimes it gets out of hand; sometimes that person gets insulted, hit or beaten up. What happens?

5. Are there any classmates who are often bullied or isolated by others? Why this happens?

6. Are there any classmates who often bully others? Describe the characteristics of children that bully others?

7. How do you think and feel when you see someone (not a friend) is being bullied? Will you do something?

8. Has anyone (parents, teachers or others) told you a certain way that they think you should do when bullying happens?

**-Questions for victims**

1. Have you ever been picked on, harassed, forced to do something against your will, and/or bullied since you’ve been in school? If yes, tell me what happened. How often has this happened to you? When did it start?

2. Tell me about the person doing this to you: Is he or she older or younger? Is he or she bigger/stronger or smaller/weaker? What about his/her academic performance?

3. What is your feeling after this happens? Or other symptoms caused by this? Has the situation had any impact on your grades?

4. How did you react? Did you seek help? From whom? Was this way helpful?

**-Questions for bullies**

1. Have you ever picked on, harassed, or forced others since you have been in school? When did this start? How often would you do this to others?

2. Tell me about the person you have ever bullied: Is he or she older or younger? Is he or she bigger/stronger or smaller/weaker? What about his/her academic performance?

3. What is your feeling after doing this?

4. Do your parents, teachers or others know this? What about their attitudes? How did they deal with this? What was the final result of this?

5. Have you ever been punished about bullying others? What kind of punishment? Who did this?

**Questions for Cyberbullying (we can talk about the movie *Shao Nian De Ni* as opening question)**

1. Does the school allow you to carry smartphones or other online devices?

2. Do you know the term “cyberbullying”, and does there anyone around you involved in such incidents? What happens? Does this have any impacts on them or their life?

3. Have you ever been involved in any of the cyberbullying situations as victims, and what happens? Please describe your feelings about this. Does this have any impacts on you?

4. Have you ever been involved in any of the cyberbullying situations as bullies, and what happens? Please describe your feelings about this. Does this have any impacts on you?

5. Does there any education or preventive measures about cyberbullying in your school?

6. what is your opinion of cyberbullying, is it common or not around you?

7. Do you know incidents personally that combines both traditional bullying and cyberbullying?

**-General questions about tackling**

1. What are the school factors that seem to breed aggressive behaviors?

2. How does the current school setting deter bullying? Does there have any rules or policies about bullying in your school?

3. What do you think of school bullying? is it common? As for school bullying and cyberbullying, which is more prevalent in your school? Which has bigger impacts on your life?
