# Supplementary material for: Experiences and Perspectives of Traditional Bullying and Cyberbullying Among Adolescents in Mainland China-Implications for Policy
Source: Front Psychol. 2021 Jul 6;12:672223. doi: 10.3389/fpsyg.2021.672223 (PMC8290073; doi:10.3389/fpsyg.2021.672223)
Supplement: Supplementary file 2 [file Table_2.DOCX]

**关于校园欺凌的问题**

**基本信息**

1. 谈谈你的家庭 (是否有兄弟姐妹、是否跟父母生活在一起、跟父亲母亲的关系).

2. 你觉得学校怎么样啊? (班级数量、每个班级的学生人数、班级纪律、跟老师们的关系、跟同学们的关系)

3. 成绩怎么样啊（排名）?

4. 你看过有同学被欺负吗，例如被骂、被恶意嘲笑、被推、被打? 具体发生了什么？

5. 班级里有同学经常被欺负或被大家孤立吗? 为什么呢?

6. 班级里有同学经常欺负别人吗? 他们为什么这么做呢？这部分同学有什么特点？

7. 当你看到有人被欺负时，你有什么感受吗，你会做些什么吗?

8. 父母、老师或其他人有跟你谈过校园欺凌相关的事件吗，有说过你应该怎么处理此类事件吗？

**被欺凌者**

1. 你在学校里被人欺负过吗（语言、身体、心理）？发生了什么，什么时候开始的，多久发生一次？

2. 欺负你的人是怎么样的？（性格，高年级的吗,比你高壮吗，成绩怎么样）

3. 这件事情的发生对你有什么影响吗？（情绪上、身体上、学习）

4. 你有什么反应或回击吗，你向他人寻求帮助了吗，向谁？有用吗，他们真的帮到你了吗？这件事情的处理结果是怎样的呢？（如果没有求助他人，那为什么不呢？）

**欺凌者**

1. 你在学校里欺负过别人吗，发生了什么？什么时候开始的，你多久会这样一次呢？

2. 你欺负过的人有什么特点吗？（性格、外貌、学习成绩）

3. 这件事发生之后，你有什么感受吗？

4. 老师、父母或其他人知道这件事吗，他们怎么说？你有因为这件事情被罚过吗？这件事情最后的处理结果是怎么样的？

5. 你曾经有过因为欺负他人而被罚的经历吗？

**关于网络欺凌的问题 (可以以电影“少年的你”开场)**

1. 学校允许你们带手机或其他可以上网的设备吗?

2. 你听说过 “网络欺凌”这个词吗？ 你周围有同学被卷入到这种事件吗? 发生了什么? 这些对于他们或他们的生活有什么影响吗?

3. 你有被卷入到网络欺凌事件中吗，发生了什么? 你有什么感受吗？卷入到类似事件中对你或你的生活有什么影响吗？

4.在你的学校，有关于网络欺凌的相关教育和预防措施吗?

5. 你认为在你的周围，网络欺凌普遍吗？

6. 在你的身边，你知道有校园欺凌和网络欺凌联合发生的情况吗？

**讨论**

1. 在你的学校有什么因素可能导致欺凌事件的发生吗?

2. 在你们学校关于欺凌有什么相关的校规校纪或规章制度吗?

3. 你觉得校园欺凌常见吗? 在你身边，你觉得校园欺凌和网络欺凌哪个更常见，哪个对你们的生活产生的影响更大?
